# Optimized robust learning framework based on big data for forecasting cardiovascular crises

**DOI:** 10.1038/s41598-024-76569-6

**Published:** 2024-11-15

**Authors:** Nadia G. Elseddeq, Sally M. Elghamrawy, Ali I. Eldesouky, Mofreh M. Salem

**Affiliations:** 1 Computers Engineering and Systems Department, Mansoura University, Mansoura, 35516 Egypt; 2 Computer Engineering Department, Misr Higher Institute for Engineering and Technology, Mansoura, 31527 Egypt

**Keywords:** Robust learning, Robust preprocessing techniques, Big Data, Forecasting cardiovascular crises, Optimization, Cardiovascular biology, Cardiovascular diseases

## Abstract

Numerous Deep Learning (DL) scenarios have been developed for evolving new healthcare systems that leverage large datasets, distributed computing, and the Internet of Things (IoT). However, the data used in these scenarios tend to be noisy, necessitating the incorporation of robust pre-processing techniques, including data cleaning, preparation, normalization, and addressing imbalances. These steps are crucial for generating a robust dataset for training. Designing frameworks capable of handling such data without compromising efficiency is essential to ensuring robustness. This research aims to propose a novel healthcare framework that selects the best features and enhances performance. This robust deep learning framework, called (R-DLH2O), is designed for forecasting cardiovascular crises. Unlike existing methods, R-DLH2O integrates five distinct phases: robust pre-processing, feature selection, feed-forward neural network, prediction, and performance evaluation. This multi-phase approach ensures superior accuracy and efficiency in crisis prediction, offering a significant advancement in healthcare analytics. H2O is utilized in the R-DLH2O framework for processing big data. The main improvement of this paper lies in the unique form of the Whale Optimization Algorithm (WOA), specifically the Modified WOA (MWOA). The Gaussian distribution approach for random walks was employed with the diffusion strategy to choose the optimal MWOA solution during the growth phase. To validate the R-DLH2O framework, six performance tests were conducted. Surprisingly, the MWOA-2 outperformed other heuristic algorithms in speed, despite exhibiting lower accuracy and scalability. The suggested MWOA was further analyzed using benchmark functions from CEC2005, demonstrating its advantages in accuracy and robustness over WOA. These findings highlight that the framework’s processing time is 436 s, mean per-class error is 0.150125, accuracy 95.93%, precision 92.57%, and recall 93.6% across all datasets. These findings highlight the framework’s potential to produce significant and robust results, outperforming previous frameworks concerning time and accuracy.

## Introduction

As per the World Health Organization (WHO), cardiovascular disease (CVD) is a severe health issue across the world, causing the death of millions each year^1^. Blood pressure (BP) is a major vital indicator evaluated in both non-clinical and clinical settings, representing the force with which blood circulates throughout the body^2–4^ BP has three states: Normotensive (90/60 to 120/80 mmHg), hypertension, or hypotension. BP readings are classified as either greater than 140/90 or less than 90/60 mmHg^2^. Both hypotension and hypertension are major risk factors for CVD. Hence, these diseases are identified and prevented through continuous monitoring of BP^3^. Continuous or intermittent BP monitoring is utilized depending on the clinical context. However, standard clinic BP machines frequently fail to acquire these values for patients experiencing ’white coat hypertension’ syndrome^5^. When compared to an in-clinic sphygmomanometer, a normal BP individual is more likely to be diagnosed with hypertension. As a result, the measurement returns to normal outside of the medical setting. This suggests that a genuine person’s BP is impacted by unique sentiments, which may be caused by acute worry. As a result, rather than monitoring BP sporadically, continuous BP monitoring is important for determining the real BP in the presence of BP variability (BPV)^6,7^. Several works of literature have investigated robust machine learning (ML) frameworks that use data from clinical trials, illness history, and other physiological features of CVD^8,9^. H2O is an open-source, rapid, and scalable DL task and advanced analytics platform that enables the development of Neural Network (NN) systems^10^. Deep learning architecture (DLA) is a hierarchical feature extraction model with numerous degrees of nonlinearity. DL models have functions such as representing the original data. It also displays the optimal result for complex data^11^. Lots of research employed various optimization algorithms to solve decision-making issues^12–14^. A meta-heuristic optimizer’s efficacy is measured by its capacity to make a compromise between the exploitation-exploration^15^. In the process of optimization, a lot of metaheuristics use early detection to accurately analyze the feasible area and prevent stagnation within local optimal.

To overcome the Feature Selection (FS) process, the scientists offered various hybrid techniques. Mafarja et al.^16^ investigates a hybrid technique combining the WOA and simulated annealing. Ding et al. and Kahya et al.^17,18^ also reports on genetic algorithm (GA) and particle swarm optimizer (PSO). It should be noted that a hybrid technique combining FS filter and wrapper approaches have already been investigated^19,20^. The main objective of using the new variant WOA continuous optimizer is to detect emergency CVD cases in intensive care units.

According to the above, there continues to be requirement for a current, robust, accurate, and fast framework to handle emergency situations, as they require more accurate and high-speed. During comparison with accuracy, the robustness of data pre-processing methods may be an essential consideration, particularly in cases where missing data is common. As a result, these techniques should be emphasized to reduce noise interference and other erroneous data. Cleaning incomplete datasets during the pre-processing phase might help to protect the integrity of the treated dataset. In this regard, the study presents a robust, rapid and precise DL framework to solve these previous issues. Our framework includes robust pre-processing methods like Borderline SMOTE (BSMOTE)^21^ and Adaptive Synthetic Sampling (ADASYN)^22^. All of these methods provide balanced data, resulting in significant performance improvements and robustness for downstream classification tasks.

The manuscript is formulated as below “Motivation & contribution” displays motivations & contributions of the paper. “Related work” reviews the related work. “The Proposed R-DLH2O Framework” displays the Modified WOA (MWOA) and proposed framework. “Experimental and result discussion” discussed the experiments. “Conclusion and future directions” displays the conclusion.

## Motivation & contribution

The motivation of the paper: first: Three frameworks were proposed in the paper^23^ for learning and predicting cancer, and under any situation the 3rd one was superior in accuracy with time to other frameworks for all types of cancer. Therefore, our DL framework is based on this framework but we are trying to improve the performance on a new type of emergency disease and using a model which process big data fast. Second: Introduce a fast accurate optimizer, namely Modified WOA (MWOA). This optimizer is essential for FS to minimize the amount of data utilized and to tweak the feed-forward neural network (FFNN). In addition, MWOA is used to tune the hyperparameters of FFNN to determine the optimal number (no.) of layers and neurons, which is why using metaheuristics in FFNN improves performance^24^.

The paper’s primary contributions include the following:

- The proposed R-DLH2O framework can handle a wide range of data types, as patient data incorporates information from a variety of sources. H2O is utilized in the R-DLH2O framework for processing big data.
- Optimizing forecast and decreasing the size of input data to FFNN by choosing optimal features using FS technique to train the neural network NN.
- The framework has been evaluated on three benchmark datasets, including Normotensive Hypertensive, Hypotensive patients to show their dependability and efficiency. These datasets are freely available to the public and are still utilized in the majority of contemporary studies^25–31^.
- The efficiency of MWOA is compared with other optimizers. This research will aid provide the most relevant, accurate and rapid framework for emergency situations because the suggested technique has been used with data acquired from hospital medical information systems.

## Related work

The continuous optimizers have been used in the literature for a variety of research purposes, including gene selection, face recognition, illness diagnosis, and FS. In^32^, Nakamura et al. carried out studies to compare various meta-algorithms, including BAT, FFA, and PSO. In^33^, Sharawi et al. created an FS dependent on the WOA and showed that the WOA can locate the optimum features with high-accuracy. In^34^, Hassan et al. show that combining WOA with Naive Bayes (NB) in a hybrid algorithm speeds up classification while saving storage capacity. In^35^, Mirjalili et al. showed the WOA’s superiority over other meta-algorithms and traditional approaches. In^36^, Elisa Meja-Meja et al. assessed the ability of characteristics generated from photoplethysmography (PPG) based Pulse Rate Variability (PRV) to identify the three blood pressure states. They also evaluated its capability to predict median arterial, systolic, and diastolic BP in critical instances. In^4^, C El-Hajj, et. al proposed different models for estimating systolic and diastolic BP by applying recurrent neural networks (RNNs) with bi-directional links and focus processes based solely on PPG data. In^37^, Latifa Nabila Harfiya et al. suggested a new DL model for learning how to translate signals from PPG to arterial blood pressure (ABP). In^38^, PEIHAO LI et al. developed two frameworks for estimating ABP at the central arteries using PPG and electrocardiogram. In the paper presented by Elghamrawy et al. in^39^, a novel hybrid nature-inspired is introduced, termed the Genetic Grey Wolf Optimization (GGWO). Hybridization of the GA and GWO is employed for addressing the early convergence and weak result exploitation issues in GWO. In^40^, Elghamrawy et al. proposed an approach called Genetic Based-Adaptive Momentum Estimation (GB-ADAM) for optimizing CNN hyperparameters. Its technique utilizes GA to improve the Adam optimizer’s setup settings, resulting in improved accuracy in classification. In^41^, Khan et al. showed the FFNNs are widely popular due to their universal approximation capability, allowing them to accurately solve complex non-linear problems that are challenging for classical statistical techniques. Theoretically, FFNNs are universal approximators, making them more effective than traditional methods for addressing complex problems. Table 1 summarizes the findings of the number of prior approaches. The specifics of the artificial neural network (ANN) employed in our approach, as well as the modified WOA used in the suggested framework, are discussed.

**Table 1:** The results of previous studies using different methods.

Ref	Year	Methods	Hypertensive (P1)			Hypotensive (P2)			Normotensive (P3)		
			mean per class	No. of features Chosen	Time in seconds	mean per class	No. of features chosen	Time in seconds	Mean Per Class	No. Of features chosen	Time in seconds
Elsayad, Ahmed et. al	^42^	AC-WOA	0.18	5	914	0.29	7	518	0.22	6	863
Mirjalili et al	^35^	WOA	0.18	4	818	0.29	8	622	0.22	8	1273
Nakamura et al	^32^	FFA	0.21	7	1282	0.30	6	860	0.26	6	1414
Nakamura et al	^32^	BAT	0.21	11	806	0.29	6	795	0.28	3	333
Nakamura et al	^32^	PSO	0.22	6	715	0.29	6	545	0.23	4	873

## The proposed R-DLH2O framework

**Fig. 1:**
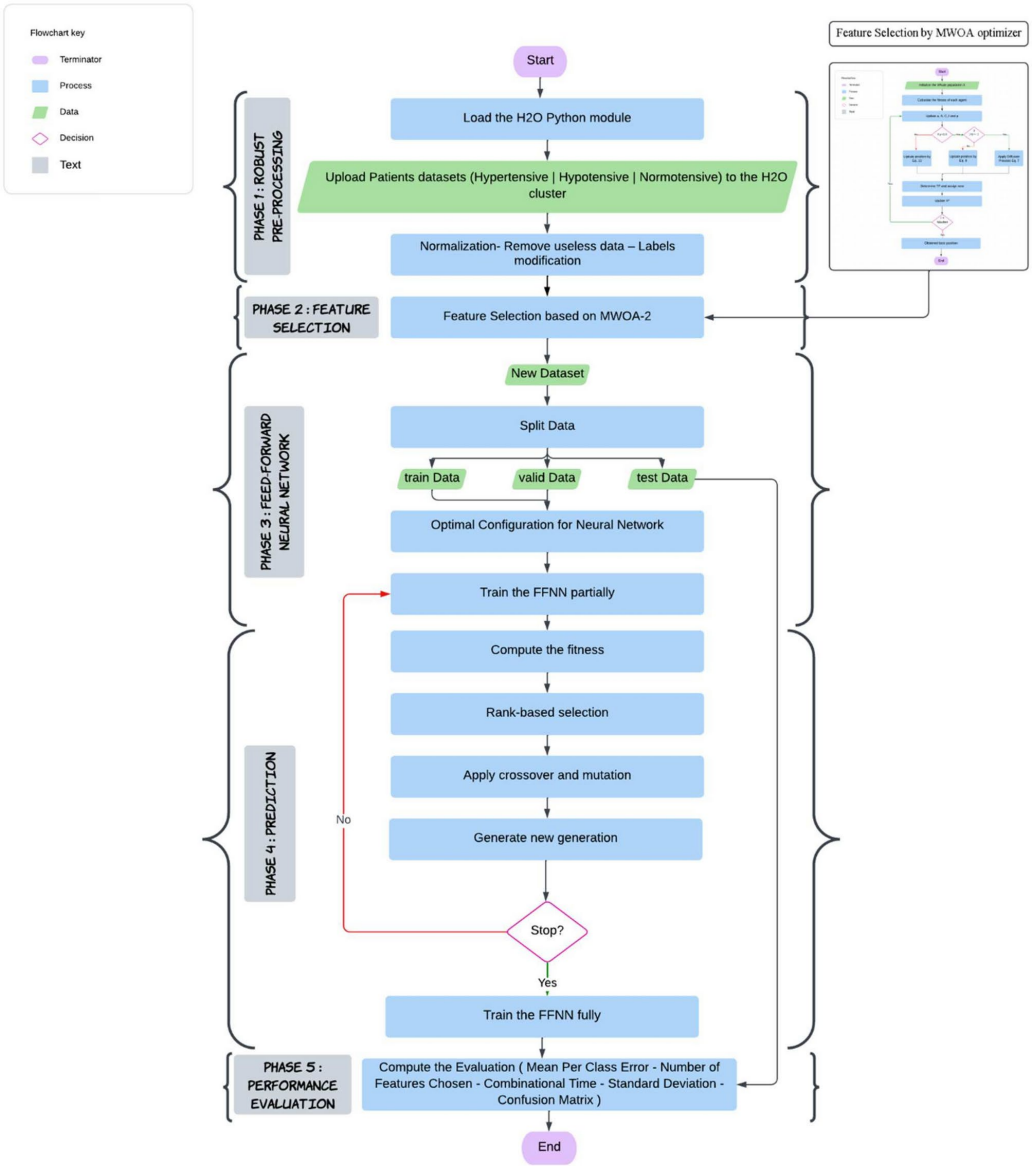
THE ARCHITECTURE OF R-DLH2O FRAMEWORK.

The objective is to suggest an R-DLH2O that has the optimal Mean-Perclass-Error (MCE) and speed for enhancing the performance of emergency CVD prediction. In^23^, Elseddeq, Nadia G., et al. proposed three DL frameworks, and, in the overall situation, the 3rd one was superior in accuracy to the others. So, our DL framework is based on an optimized parameter FFNN. The best collection of FS serves as its input. WOA was modified for this purpose. H2O is utilized in the R-DLH2O framework for processing big data. The R-DLH2O consists of 5 phases: robust pre-processing, feature selection, feed-forward neural network, prediction, and performance evaluation phases (See Figure 1).

### The robust pre-processing phase

Robust preprocessing techniques address data anomalies like outliers, missing values, and noise to enhance model reliability and performance. For the R-DLH2O framework, using methods such as data cleaning, preparation, normalization, and balancing ensures a resilient training dataset. The aim is to make preprocessing less sensitive to anomalies, leading to more accurate models.

### Cleaning and missing the data

Once the data is gathered, it may form an incomplete dataset, which is then categorized into three subsets.

The first pertains to hypertensive patients, the second to hypotensive patients, and the third to normotensive patients. Each of these subsets spans one year for every individual. Next, we have the option to clear or rebuild the dataset to ensure the integrity of the processed data and its compatibility with the R-DLH2O framework. Initially, we can clean the dataset by deleting extraneous columns, like the ‘id’ column. Subsequently, we can rectify any missing using mathematical expressions:

1 $$\mu i=\frac{{\mu }_{i-1}+{\mu }_{i+1}}{2}, i\in N$$

where ‘µi’ = missing value, ‘µi−1’= value that came before absent value, and ‘µi+1’= subsequent value after absent value. Here, ‘N’ = set of natural numbers.

Using this mathematical expression greatly improves the performance of R-DLH2O because it is useful for sequential data, making the classifier more robust in dealing with missing data, providing more robust solutions for diverse and complex data situations. This means that robust preprocessing is better than normal preprocessing alone.

### Data normalization

The empirical demonstration of the efficacy of data normalization in improving convergence rates and fostering training stability is outlined in^43^. As a result, its incorporation as a crucial pre-processing stage is imperative for the current study. In order to organize and constrain the data within a given range, we employ the widely used normalization approach known as minimum-maximum normalization. It is preferred for its ease and efficacy. This method adjusts the data to a range between 0 and 1, ensuring uniformity for analytical purposes. Below, you will find the normalization equation for variables:

2 $$DN= \frac{\left(\left|\beta \right|\right)-\left({10}^{w-1}\right)*(|\varepsilon |)}{{10}^{w-1}}$$

Let ‘β’ = data element, ‘w’ = no. of digits in element A, ‘ε’ = first digit of data element A, ‘DN’ = scaled value [0-1]

Using minimum-maximum normalization has significantly improved the performance of R-DLH2O because it is useful for scaling, making the classifier more robust to data problems, including outliers, providing better model performance. This means that robust preprocessing is better than normal preprocessing alone.

### Data imbalance

An imbalance of features in a classification scenario occurs when certain features are significantly underrepresented. Addressing this issue involves employing methods categorized into two subgroups: under-sampling and over-sampling. The random under-sampling boost (RUS) has been enhanced to address data imbalance by incorporating the AdaBoost method. For counteract the disparity and enhance ML efficiency, we implemented the BSMOTE and ADASYN methods to achieve balanced data^44,45^. These techniques are depicted in Algorithm-1 and Algorithm-2.

Using BSMOTE or ADASYN significantly improved the performance of the R-DLH2O when dealing with imbalanced datasets, making the classifier more robust to class imbalance. This means that robust preprocessing is better than normal preprocessing alone. Because it is insufficient to address these issues because it does not directly address the class imbalance problem.

During the final step of the robust pre-processing phase, we rescaled the values in the columns and transformed the labels from text format to integers. Normal, warning, alert, and emergency classifications were given values 1, 2, 3, and 4, accordingly.

### The feature selection phase

**WOA:** The researchers^35^ presented a new novel optimizer called WOA. This algorithm outperforms several existing ones.

**Algorithm-1:**
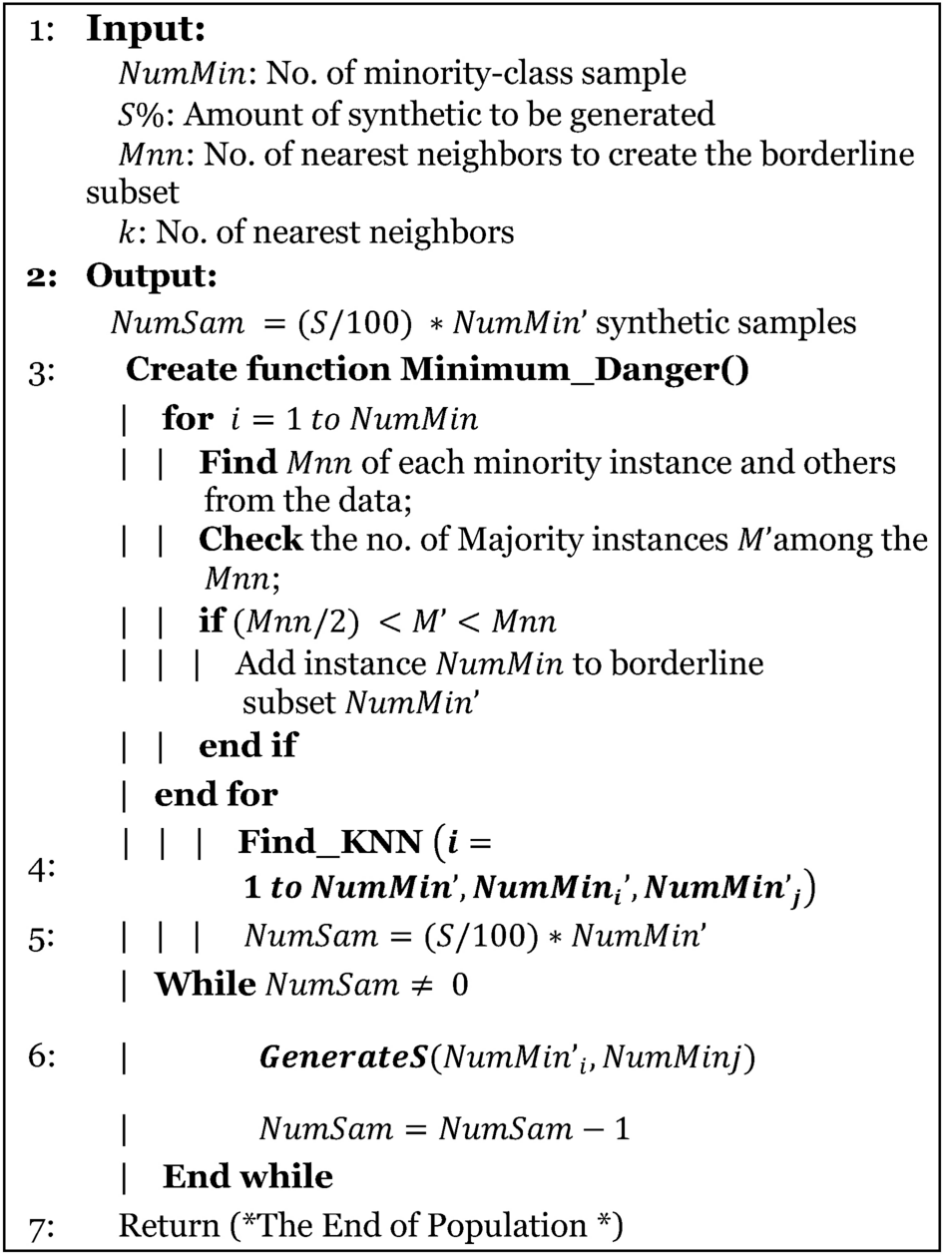
BSMOTE

**Algorithm-2:**
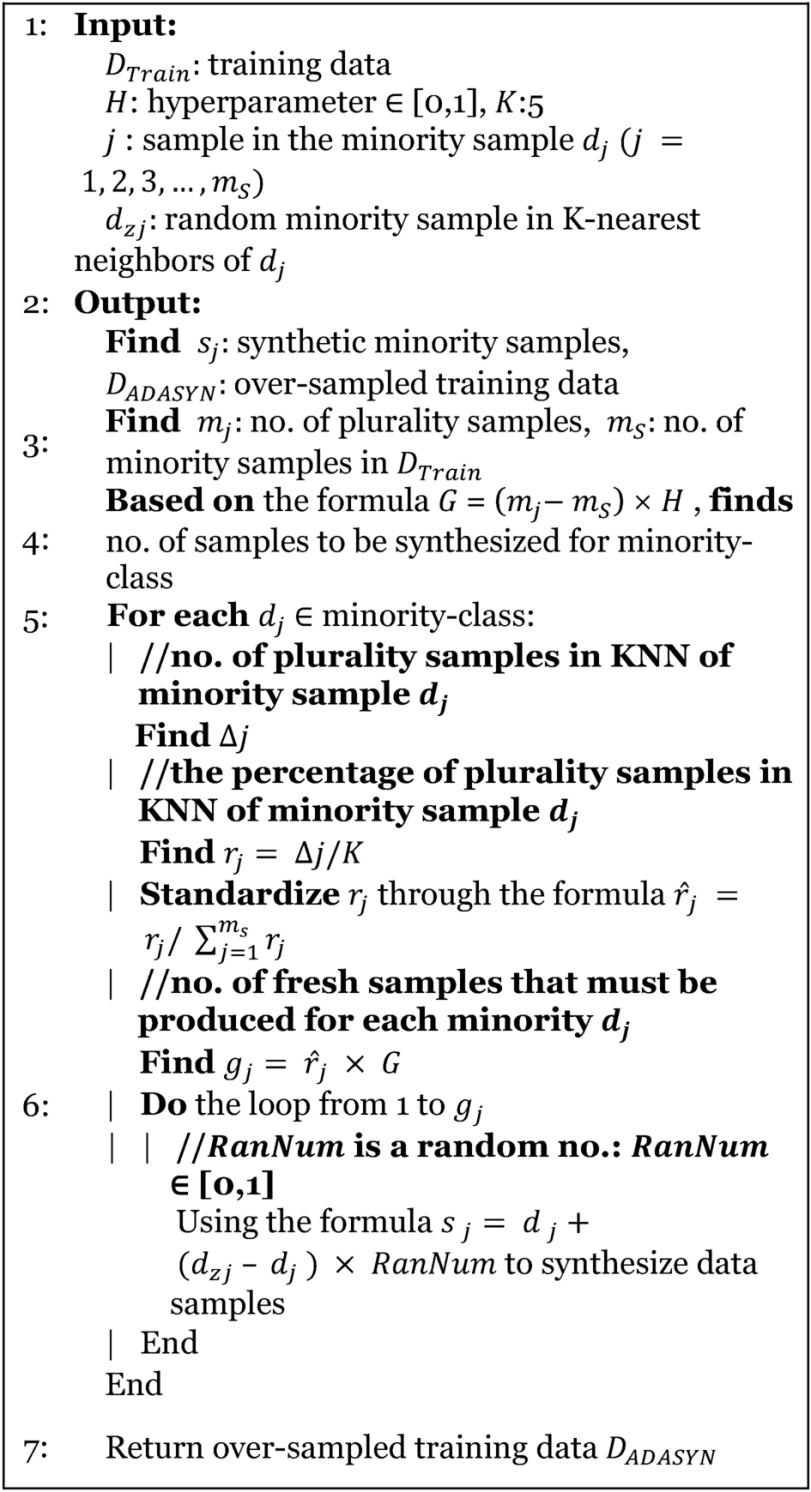
ADASYN

**Encircling prey:** The process begins by finding optimal result, that serves as the intended outcome. As a result, other adjustment locations that align with the top solution are identified. The equations for these actions are as follows:

3 $$\overrightarrow{D}= \left|\overrightarrow{C }.{\overrightarrow{X}}^{*}\left(j\right)- \overrightarrow{X}\left(j\right)\right|$$

4 $$\overrightarrow{X}\left(j+1\right)= {\overrightarrow{X}}^{*}\left(j\right)- \overrightarrow{A} .\overrightarrow{D}$$

where j represents iteration, $\overrightarrow{A}$ and $\overrightarrow{C}$ = vectors, and $\overrightarrow{X}$* = placement vector of best-result. $\overrightarrow{A}$ and $\overrightarrow{C}$ measure with:

5 $$\overrightarrow{A}=2\overrightarrow{a} . \overrightarrow{{r}_{1}}-\overrightarrow{a}$$

6 $$\overrightarrow{C}=2 . \overrightarrow{{r}_{1}}$$

Where $\vec{r}$ = vectors in [0–1]. During iterations, we need to reduce $\overrightarrow{{\varvec{a}}}$ linearly from [2–0].

**Exploitation phase:** This phase is divided into two parts:

- Shrinking updating: This was accomplished to lower the quantity of $\overrightarrow{a}$ in (5). A whale’s new locations could be described as being between the original and present ideal placements.
- Spiral updating: The range from a whale to a target may be measured:

7 $$\vec{X}\left( {j + 1} \right) = \vec{X}^{*} \left( j \right) + \vec{D}^{{ {^{\prime}}}} \times e^{wl} \times \cos \left( {2\pi l} \right)$$

8 $$\vec{D}^{ ^{\prime}} = \left| {\vec{C}.\vec{X}^{*} \left( j \right) - \vec{X}\left( j \right)} \right|$$

= $let\,\vec{D}^{\prime}$ hole between best-result for the group & present state of the individual whale. Constant w describes a logarithmic spiral form. ‘I’ takes a value in [− 1, 1]. The mathematical model for transitioning among both of these methods is:

9 $$\overrightarrow{X}\left(j + 1\right)=\left\{\begin{array}{c}{\overrightarrow{X}}^{*}\left(j\right)-\overrightarrow{A} . \overrightarrow{D} , h<0.5\\ {\overrightarrow{D}}^{ {\prime}}{e}^{wl} . cos(2\pi l))+{\overrightarrow{X}}^{*}(j) , h\ge 0.5\end{array}\right.$$

where h = a value in [0, 1].

### Exploration phase:

**Fig. 2:**
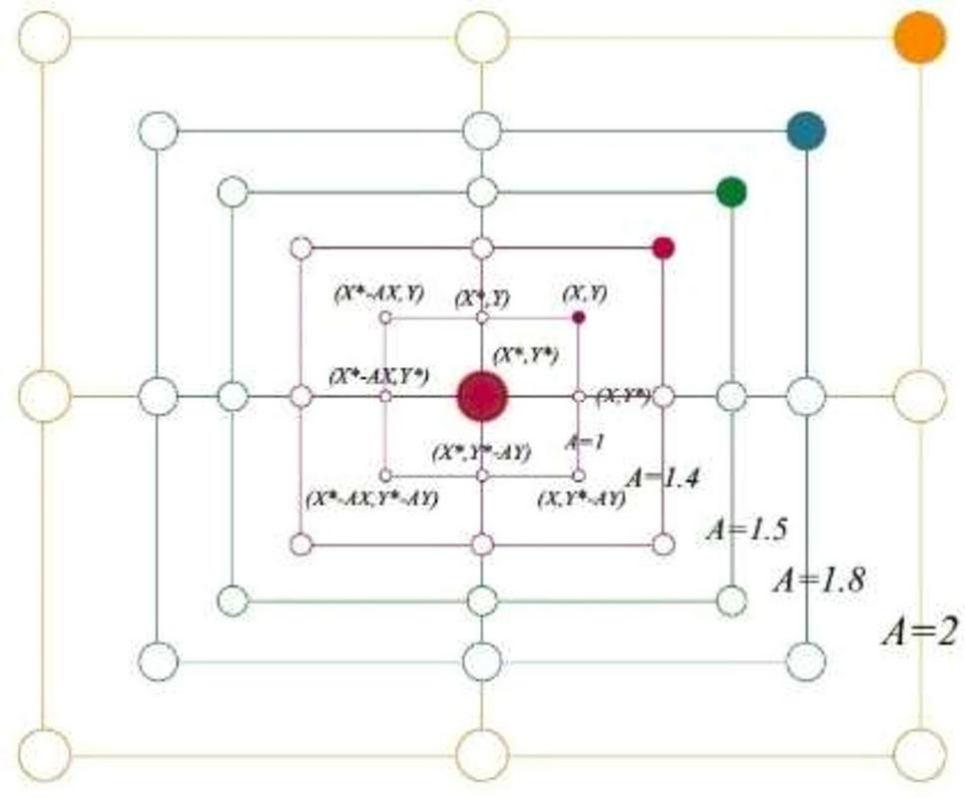
The exploration phase^35^.

$\overrightarrow{A}$ = a value [-1,1]. The specific job of the whale is identified by picking an agent that permits (See Fig. 2).

10 $$\overrightarrow{\text{D }}= |\overrightarrow{\text{C}}\cdot {\overrightarrow{\text{X}}}_{\text{rand}} - \overrightarrow{\text{X}} |$$

11 $$\overrightarrow{\text{X}}\left(\text{j }+ 1\right) = {\overrightarrow{\text{X}}}_{\text{rand }}- \overrightarrow{\text{A}} . \overrightarrow{\text{D}}$$

let $\overrightarrow{X}$_rand_ = location vector generated at random.

In a DL framework, FS is generally not required because the NNs can automatically identify and learn the most relevant features from the raw data. However, during this stage, a recent version of the WOA called MWOA is employed to address the problem of FS. The dataset initially contains a total of 2N features, providing a wide range of features to investigate for reducing the feature count. Incorporating FS into the model development process for dimensionality reduction is essential. By reducing the no. of features, we can decrease model complexity, resulting in faster training times and reduced computational resource requirements. Furthermore, by selecting the fewest no. of features, the optimal feature combination is achieved, leading to a minimum error-rate. As the solution improves, the no. of features used decreases, enhancing the model’s efficiency and performance

12 $$FitnessFunction = \frac{1}{{\varvec{N}}}\boldsymbol{ }\sum_{{\varvec{k}}=1}^{{\varvec{N}}}{\varvec{M}}{\varvec{C}}{\varvec{E}}({\varvec{K}})$$

A learning technique is repeated ‘N’ times. The fitness function = the mean of the MCE. The key objective here is to minimize the resulting value.

### Feed-forward neural network phase

**FFNN:** The human brain system is based on the FFNN. The links among these nodes do not constitute a loop^46^. It is employed in a variety of systems. The neuron is the most important component; nonetheless, data travels numerically from ‘$s$’ neurons in a previous stage to neuron ‘$m$’ and flows across as an accumulation:

13 $${Z}_{m}=b(\sum_{n=1}^{s}{q}_{mn }{x}_{mn}-{c}_{m0})$$

where ${q}_{mn}$= weight of communication between neuron ‘$n$’ of preceding stage to a current neuron ‘$m$’. ${x}_{mn}$= data sets and ${c}_{m0}$= established threshold for ‘m’ is used as specific weight.

**Fig. 3:**
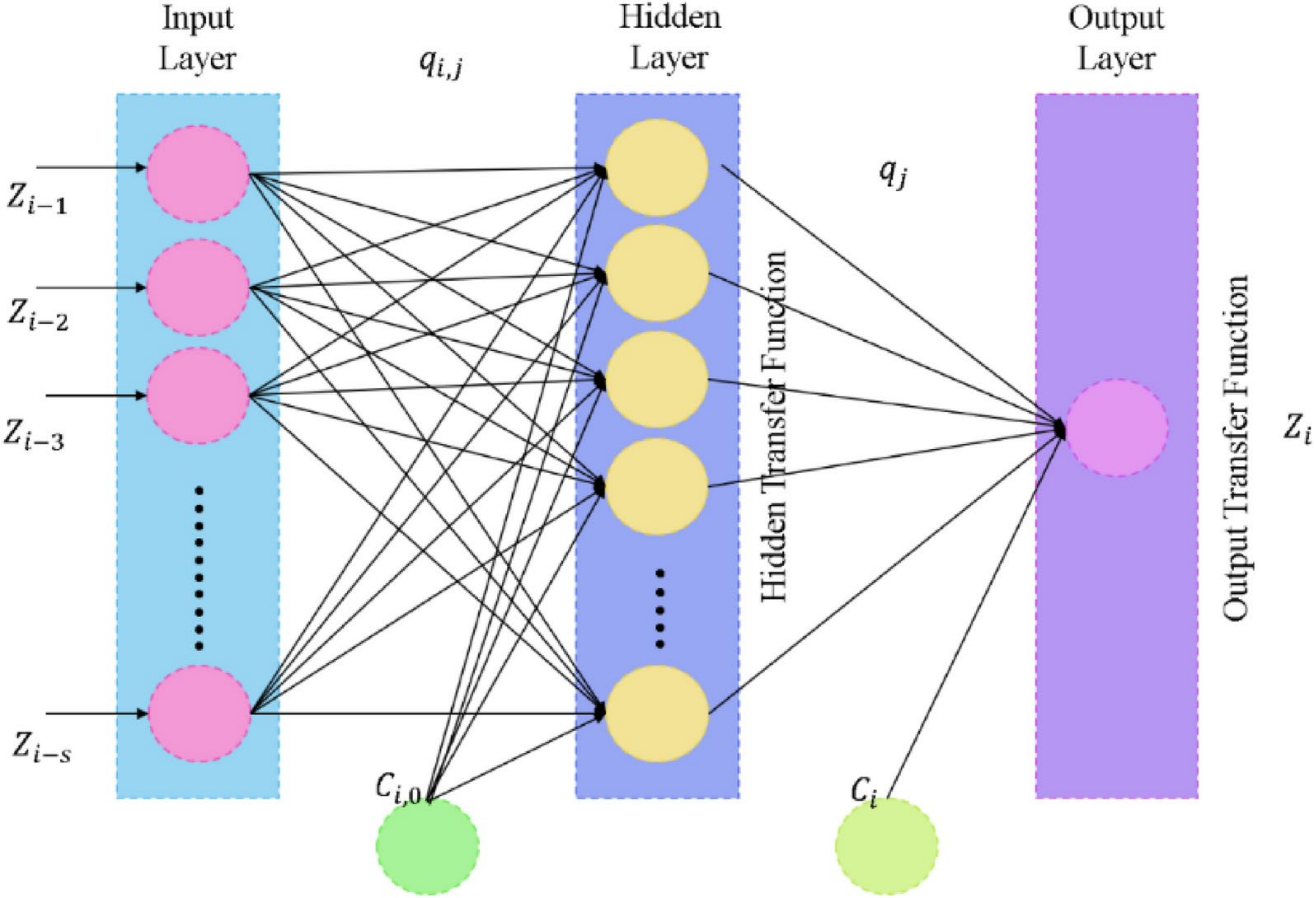
Ffnn model.

Adapt hidden or output neurons using the ‘$X$’ activation function (see Table 2). A NN contents from 3 layers: input, hidden, and output, with neurons in the input & output layers denoted by ‘$s$’ (Fig. 3).

**Table 2:** Activation functions.

Name	Activation function
Linear	${\text{X}}\left( \eta \right) \, = \, \eta$
Sigmoid	$X(\eta ) = \frac{1}{{1 + e^{ - \eta } }}$
TanH	$X\left(\eta \right)= \frac{{e}^{\upeta }-{e}^{-\upeta }}{{e}^{\upeta }+ {e}^{-\upeta }}$
Step	$${\text{X}}\left( \eta \right) \, = \begin{array}{*{20}c} { - 1\,\,if\,\eta < 0} \\ {1\,\,if\,\, \ge 0} \\ \end{array}$$

An objective was to find an FFNN weight that accurately captures a connection between the (Input/ Output) vectors^47^. Using the following equation can reduce the execution work for the pattern:

14 $$W= \frac{1}{2Z}\sum_{z=1}^{Z}\sum_{g=1}^{G}{({y}_{g}^{z}-{d}_{g}^{z})}^{2}$$

where ‘$W$’ is total mean sum error2 between the observed results. y^z^ = genuine situation. and d^z^ = intended configuration. Both ‘$z$’, ‘$g$’ represent the values for a z^th^ represent training and gth part of a result vector.

In our study, we chose a FFNN over RNNs like Long Short-Term Memory (LSTM) or Gated Recurrent Unit (GRU) for forecasting because FFNNs offer a simpler and faster training process. Given the specific nature of our forecasting problem, where the temporal dependencies are adequately captured by the FFNN, the reduced computational complexity of the FFNN provided a practical advantage without compromising model effectiveness.

The third phase is made up of FFNN that has been optimized. This signifies that MWOA has determined its settings to be optimal. These settings, such as activation function, no. of layers & neurons, and biases, of TanH, 3, 10, and random. In terms of initial weights, H2OFrame IDs set the weights to the default initial weight distribution. The training parameters, such as the learning= rule Levenberg–Marquardt and sum error^2^ = 0.01, are specified. MWOA, WOA, AC-WOA^42^, FFA, BAT, and PSO optimizers are tested in the phase. Following the optimization process, each optimizer delivers the optimum solution, which is validated using test data. Various parameters were gathered for comparison throughout the previous testing procedure.

### Prediction phase

The MWOA is employed to pick a solution for an optimization issue, involving both exploitation and exploration.

### Performance evaluation phase

This phase presents a detailed performance research of the proposed approach R-DLH2O over H2OFrame using 4 various evaluation metrics: MCE, no. of selected features, Combinational time, and Standard Deviation (STD). “Experimental results and discussion” describes these metrics in detail.

### Modified whale optimization algorithm (MWOA)

WOA is an extremely effective meta-optimizer for exploration and exploitation. Many recommendations, such as altering the exploration equations, have been explored to help with the procedure for updating, the parameters, but the problem is very difficult^48^.

#### Modifying the parameters (MWOA-1)

In exploration and exploitation, various parameters are employed to modify the algorithm. Some of these parameters have a significant influence on efficiency. Multiple versions of the algorithm exist that can achieve better efficiency. Similarly, scientists gave little thought to the readability of the typical WOA.

This work focuses on tuning variables in exploration and exploitation. The recommended changes to ‘$a$’, ‘$a2$’, ‘$A$’, and ‘$C$’ may have an impact on the two stages of WOA. These modifications are:

15 $$a=2-\text{cos}r\times \frac{(j-1)}{(Max\_iter -1)}$$

16 $$a2=-1-\text{cos}r\times \frac{(j-1)}{({Max\_iter-1)}^{2}}$$

17 $$A=2\times a\times \mathit{sin}r1-a$$

18 $$C=2\times \mathit{sin}r2$$

The ‘$a$’ and ‘$a2$’ represent time variations. They are used to gradually restrict the scope of change while changing the fraction to the square of the max number of repetitions. After the change in ‘$A$’ and ‘$C$’ is changed into a sine function, the volatility of a transition becomes more effective than typical randomness (See Algorithm-3).

#### Modifying exploration equations (MWOA-2)

The Gaussian equation serves to enhance the chances of identifying global minima while preventing the trap in local minima. The DLA growth procedure employs a random walk mechanism to generate new particles, using the Gaussian distribution method. To determine an optimal response, a succession of constructed diffusion methods might be applied.

19 $$\overrightarrow {{B_{l} \prime^{*} }} = Gaussian\left( {\mu_{{\overrightarrow {{B^{*} }} }} ,\sigma } \right) + \left( {\lambda \times \overrightarrow {{B^{*} }} - \lambda \times \overrightarrow {{b_{l} }} } \right)$$

**Fig. 4:**
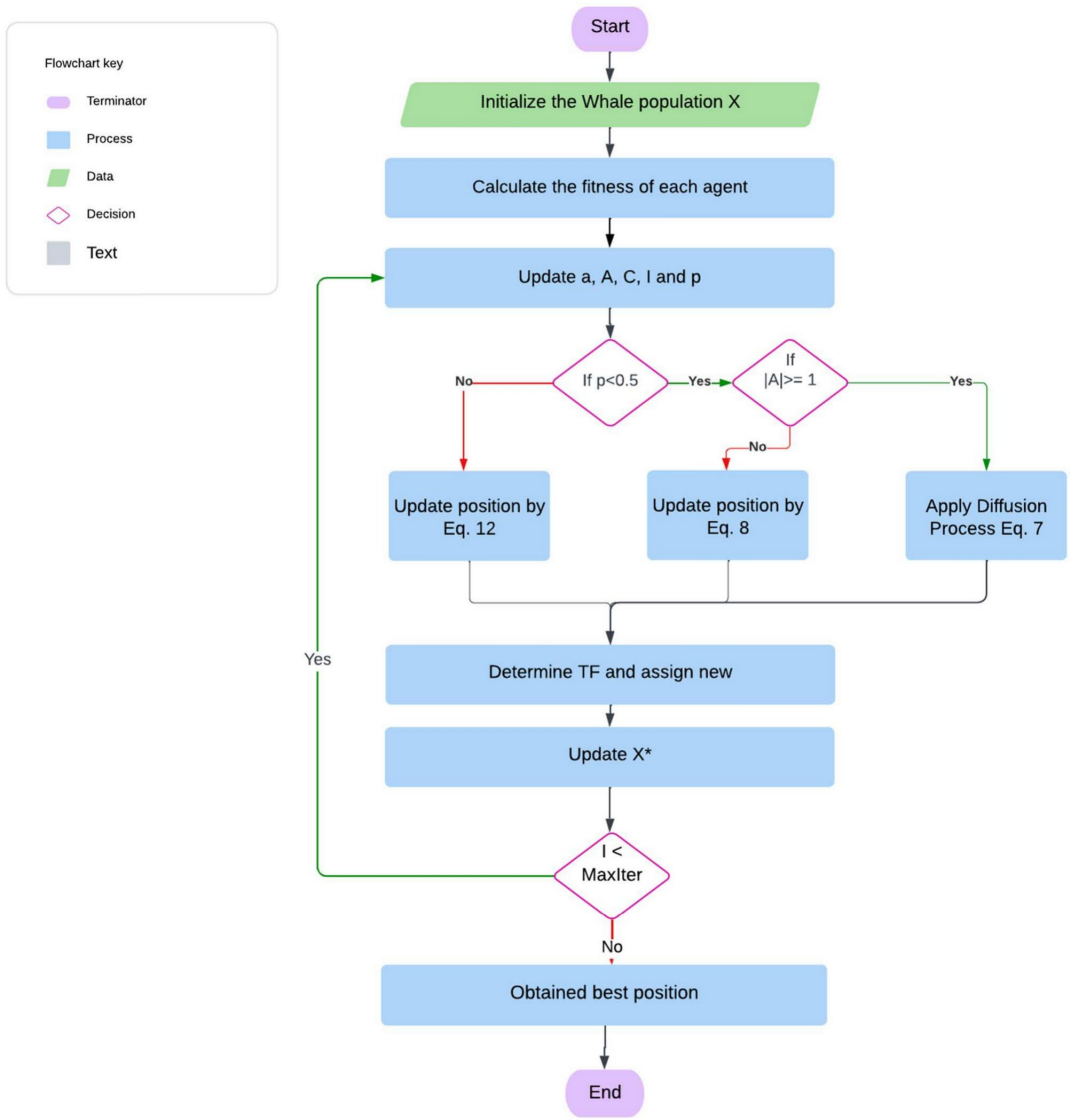
MWOA flowchart.

Where $\overrightarrow{{B}^{*}}$ represents the most effective updated diffusion method–based solution. ‘λ’ and $^{\prime}\hat{\lambda }^{\prime}$ are the random no. parameters in [0;1]. $\overrightarrow{{B}^{*}}$ and $\overrightarrow{{b}_{l}}$ indicate the more suitable placement and i-th point in the group. ${\upmu }_{\overrightarrow{{G}^{*}}}$ & σ = $\left|\overrightarrow{{b}_{l}}- \overrightarrow{{B}^{*}}\right|$ as no. of generations decreases about an improved approach. In^48^, Ibrahim, A., et al. improved the exploration potential in the suggested WOA by incorporating a diffusion procedure to find the best solution. The MWOA is an improved version of the WOA. One solution to this type of disadvantage is to use an enhanced approach to replace the search strategy for the Exploration Group. The diffusion process may be employed to generate a series of random walks around the ideal solution, expanding the WOA exploration capability to achieve best possible response. The WOA is adapted (MWOA) to improve exploration performance by employing the process of diffusion rather than the search space method in WOA. Individuals may require to continue investigating an intriguing area inside search region to prevent local stagnation, as indicated in Fig. 4, Algorithm-4.

#### Modifying the parameters with exploration equations (MWOA-3)

In this modification (MWOA-3), the previous two modifications are merged.

**Algorithm-3:**
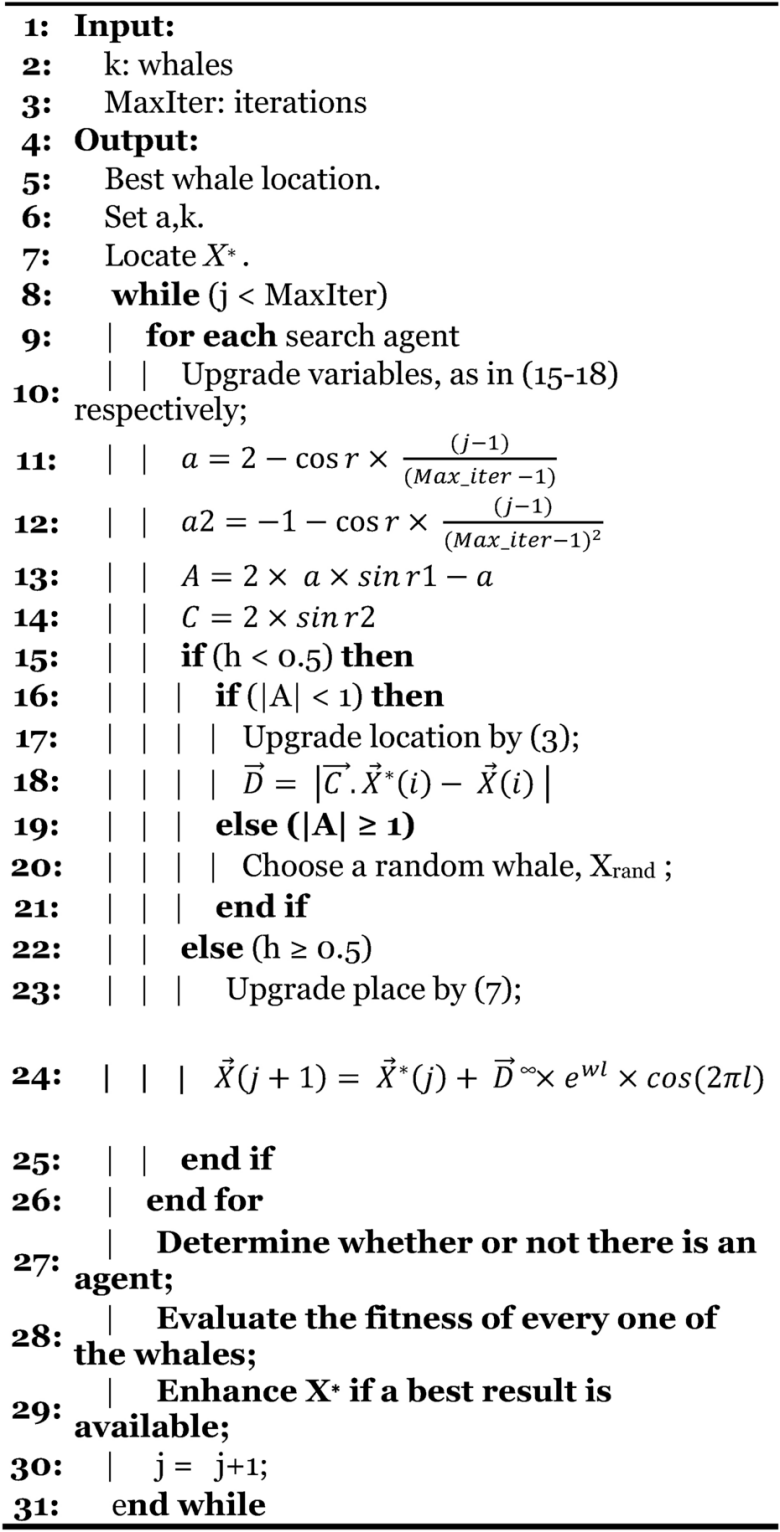
MWOA-1

**Algorithm-4:**
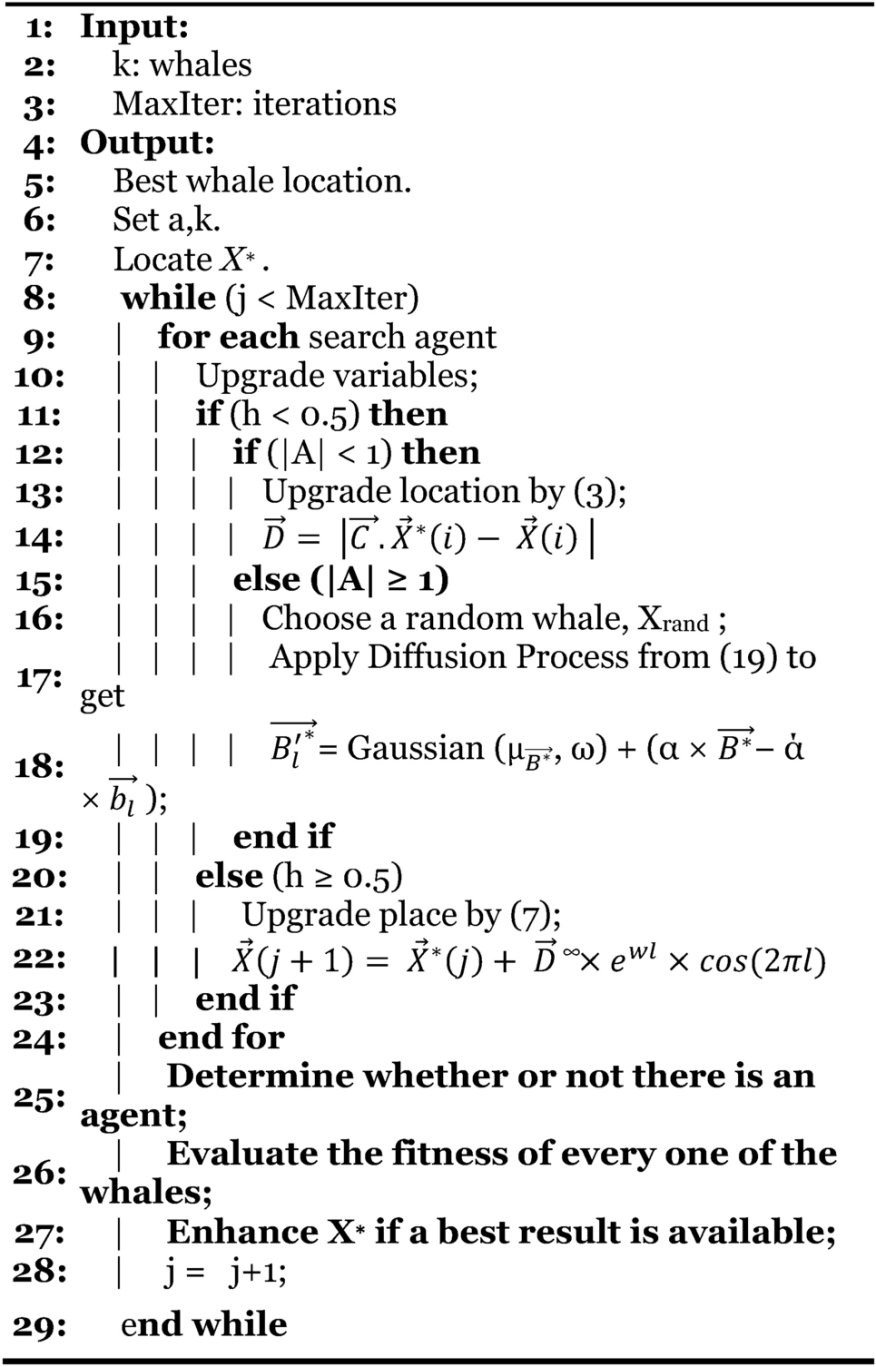
MWOA-2

## Experimental results and discussion

The paper divides the experimental section into six experiments. All testing was performed on an Intel Core i7 2.90 GHz processor, 32 GB of RAM, and an NVIDIA Quadro M2000M graphics card. The results will be obtained from various datasets selected for experimentation. As illustrative examples of problems that the MWOA can handle, these data are chosen to contain a variable number of features, classes, and instances.

The software used is Python, and the libraries used are Pandas, Time, NumPy, H2O, and H2O.estimators.deeplearning. They are applied for prediction and visual depiction of outcomes.

### Dataset description

Every test is carried out using three benchmark datasets derived from PhysioNet MIMIC-II, focusing on elderly individuals with BP problems as shown in Table 3^49^. In^50^, M. Saeed et al. reported three datasets representing cases of patients with different categories of BP disorders, containing 35,233 samples: ‘P1’ for Hypertensive, ‘P2’ for Hypotensive, and ‘P3’ for Normotensive. Their no. of features = 11, and their no. of classes = 4.

**Table 3:** Patients’ records.

	P1	P2	P3
Patient record	A42544	A42555	A42588
Female/Male	Female	Male	Female
Age	69	66	78
Illness Category	Hypertension	Hypotension	Normotensive
Monitoring start date	31–08-2012	22–06-2010	15–05-2015

Each dataset’s occurrences were randomly classified into three equal groupings. In cross-validation, these groups are referred to as follows: training set comprising 70%, testing set with 5%, and validation set with 10%.

### Experiment 1

**Fig. 5:**
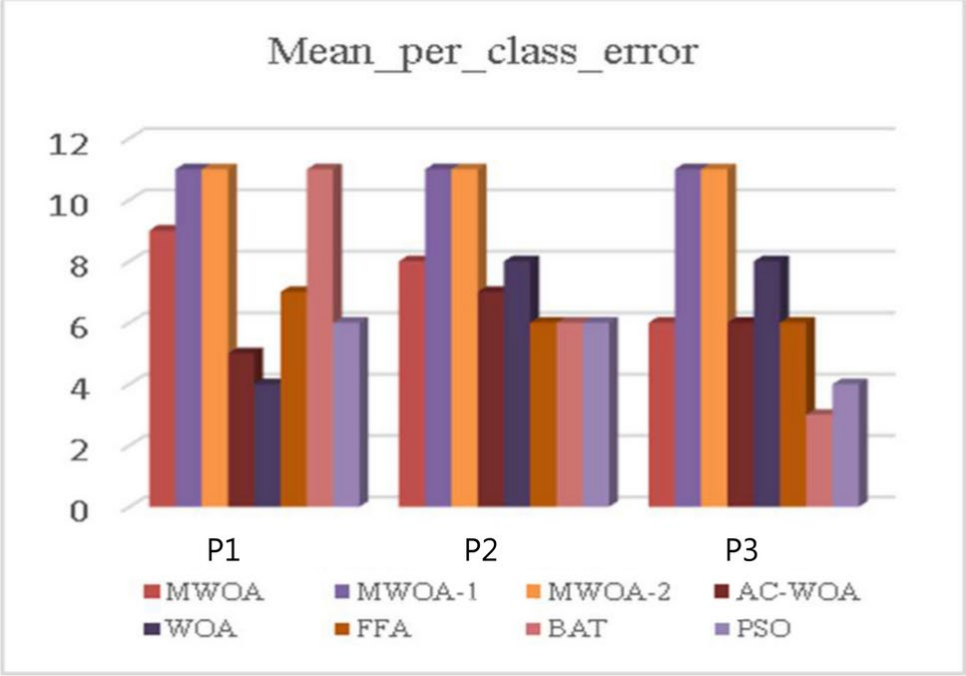
The mean_per_class_error.

The proposed MWOA-1, MWOA-2, and MWOA-3 continuous optimizers are tested against AC-WOA, WOA, FFA, BAT, and PSO to assess their performance. This procedure is repeated 50 times to achieve a stable and excellent outcome. The optimizing algorithm generates integers ranging from 0 to 2047. The trials were repeated ten times, and the mean was used to calculate a fitness score. The matrices include finding the MCE. The outcomes are described in Fig. 5 and Table 4. MWOA-2 outperforms the others for all datasets.

**Table 4:** A Mean_per_class_error comparison between optimizers.

	MWO-1	MWOA-2	MWOA-3	AC-WOA	WOA	FFA	BAT	PSO
P1	0.165	**0.129**	0.167	0.18	0.18	0.21	0.21	0.22
P2	0.242	**0.209**	0.284	0.29	0.29	0.30	0.29	0.29
P3	0.195	**0.114**	0.120	0.22	0.22	0.26	0.28	0.23

Significant values are in bold.

### Experiment 2

**Fig. 6:**
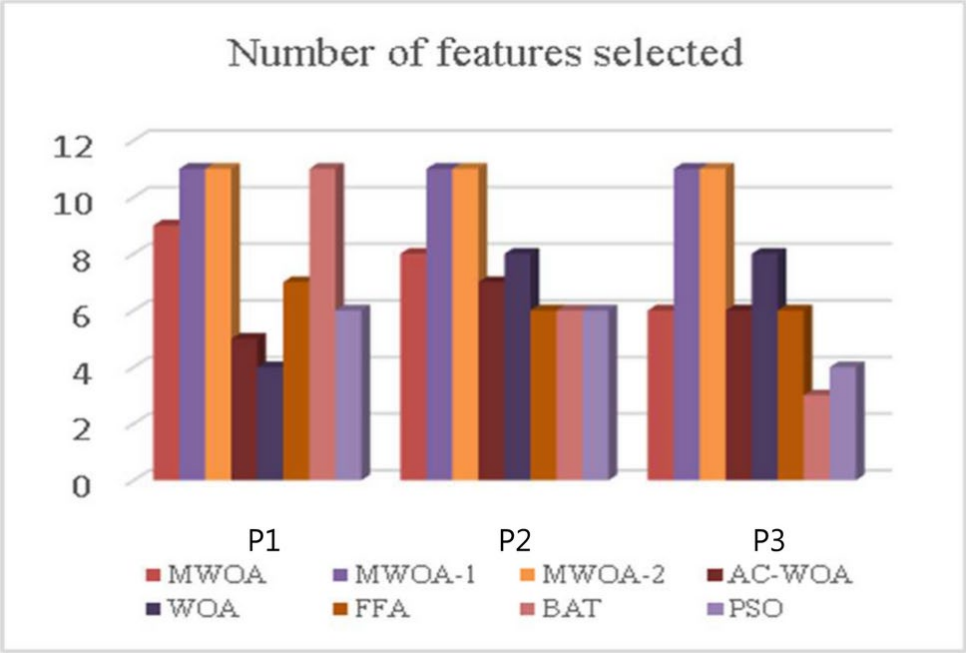
No. Of Feature selected.

The test compares the selected no. of features using proposed continuous optimizers and others on the same patient’s datasets. The outcomes are described in Fig. 6 and Table 5. Where the bar represents the no. of features selected by different algorithms. MWOA-2 selects an acceptable no. of features, unlike MWOA-1, MWOA-3, and BAT, which have selected all 11 features. (See Table 3)

**Table 5:** The no._of_features_chosen comparison by optimizers.

	MWO-1	MWOA-2	MWOA-3	AC-WOA	WOA	FFA	BAT	PSO
P1	11	**9**	11	5	4	7	11	6
P2	11	**8**	11	7	8	6	6	6
P3	11	**6**	11	6	8	6	3	4

Significant values are in bold.

### Symptoms

The variability in FS among algorithms is influenced by the differences in the characteristics of the patient datasets used for testing and the inherent characteristics of each algorithm. These factors affect how each algorithm performs and selects features.

### Experiment 3

This test compares the time required by each optimizer, and Table 6 summarizes the findings. Time and accuracy are the main factors in predicting CVD emergencies. The MWOA-2 exhibits a unique combination of timing and accuracy, making it superior to other optimizers.

**Table 6:** Different optimizers’ combinational time in seconds.

	MWO-1	MWOA-2	MWOA-3	AC-WOA	WOA	FFA	BAT	PSO
P1	515	**343**	511	914	818	1282	806	715
P2	250	**167**	228	518	622	860	795	545
P3	1195	**798**	1089	863	1273	1414	333	873

Significant values are in bold.

### Experiment 4

In this section, the MWOA is tested against standard whale, SSA, GWO, WDO, PSO, DE, and WOA versions (AC-WOA, WOABAT, WOASAT) algorithms to verify its performance. CEC’2005 benchmark functions^35^ are utilized here. For the 23 benchmark functions, unimodal F1-F7 are mostly used to validate accuracy and convergence rate. The multimodal functions F8-F13 are mainly utilized to test global search stability. Finally, fixed-dimensional multimodal functions F14-F23 are mostly applied for provide the required no. of design parameters and can offer a distinct search space.

For each function, this comparison is tested 30 times. The matrices obtain mean of all optimal values, furthermore, standard deviation (STD). If data points are far from each other, the standard deviation will be large; otherwise, it will be small^33,51^. it is:

20 $$STD = \sqrt {\frac{{\sum\nolimits_{i = 1}^{n} {\left( {x_{i} - \, x} \right)^{2} } }}{n - 1}}$$

**Fig. 7:**
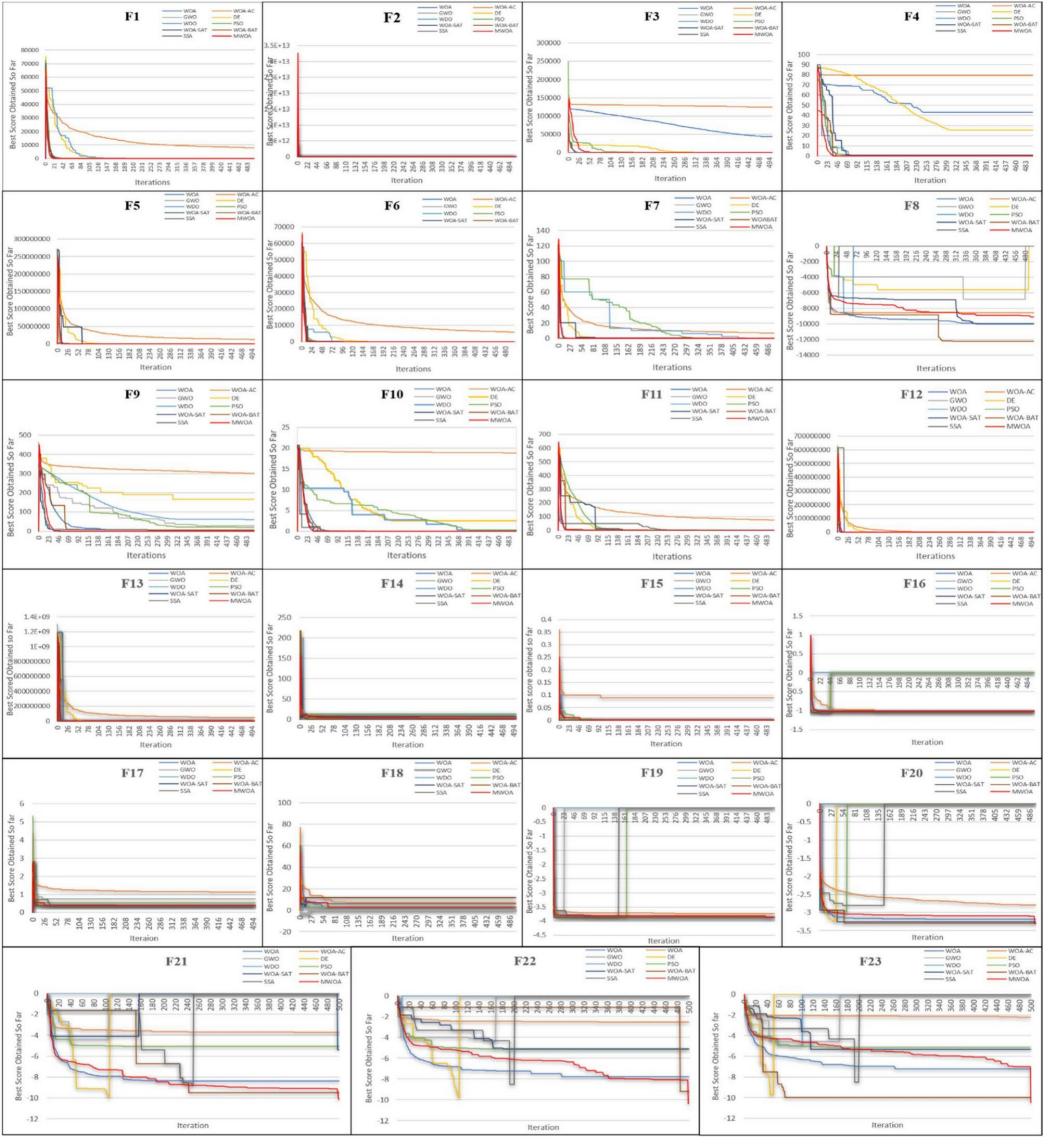
a convergence curve for the benchmark functions.

The starting parameters of the MWOA-2 are selected after extensive testing to ensure a fair comparison. During the simulation processes, alternative values for certain functions were evaluated and adjusted to obtain the most reasonable starting values for these settings. These parameters are chosen to provide an optimal solution and the shortest possible computational time, efficiently solving the issue. According to the simulation process results, the search_agent = 30, and the maximum_iteration = 500. Table 7 shows the other values for these settings, sourced from existing literature in the field, specifically from the following refs.^16,34,35,42,52–55^. The statistical findings are presented in Tables 8–10. Figure 7 indicates convergence curves of benchmark functions for competitive optimization algorithms.

**Table 7:** control parameters setteings.

Method	Settings	Value
MWOA	Convergence_factor (a) Probability_coefficient (*p*)	[0, 2] 0.5
WOA^3,5^		
AC-WOA^4,2^		
SSA^52^	Probability_coefficient (*p*)	0.5
GWO^53^	A	2 to 0
WDO^54^	RT_coefficient Gravitational_Constant Constants in the update eqcoriolis effect Maximum allowed speed	3 0.2 0.4 0.4 0.3
PSO^1,6^	Cognitive_factor (c_1_) Social_factor (c_2_) Inertia_weight (w)	2 2 0.9~0.2
DE^55^	Mutation_operator (*F*) Crossover_probability (*cr*)	0.5 0.3
WOABAT^34^	Convergence_factor (a) Probability_coefficient (p) Pulse_rate (r) Sound_loudness (Ai)	[0, 2] 0.5 0.5 0.5
WOASAT^1,6^	Reduction_rate (a) Initial_temp (t0) Max_Iter Convergence_factor (a) Probability_coefficient (p)	0.99 0.1 30 [0, 2] 0.5

**Table 8:** Compared of MWOA and others for use on unimodal issues.

Function	Metric	MWOA-2	WOA	AC-WOA	SSA	GWO	WDO	PSO	DE	WOABAT	WOASAT
F1	avg	0	7.91E–74	7.85E+03	6.60E-25	3.03E-08	0.107	5.44E-02	3.47	1.57E-06	0
	std	0	2.72E-73	5.44E+03	2.49E-24	4.81E-08	0.106	9.41E-03	10.8	7.11E-07	0
F2	avg	0	1.22E-51	6.88E+03	2.64E-14	2.00E-05	0.642	1.07	8.92E-02	7.33E-03	0
	std	0	3.36E-51	1.73E+04	1.35E-13	1.46E-05	0.860	0.187	0.311	1.39E-03	0
F3	avg	0	4.40E+04	1.25E+05	3.68E-13	0.419	0.281	0.518	6.71E+02	9.56E-06	0.793
	std	0	1.14E+04	6.04E+04	2.04E-12	0.527	0.190	0.144	3.24E+02	2.17E-06	0.500
F4	avg	0	42.9	79.5	4.79E-15	0.156	5.06E-02	0.144	25.4	1.01E-03	0.804
	std	0	26.6	5.82	2.48E-14	0.113	8.68E-02	2.63E-02	6.88	8.71E-05	0.392
F5	avg	28.6	28.8	1.28E+07	1.39E-29	28.7	35.6	33.5	1.70E+04	6.58	29.6
	std	0.213	0.458	3.71E+07	4.33E-29	0.255	9.32	1.57	7.58E+04	11.9	19.0
F6	avg	1.35	0.411	5.88E+03	3.35E-19	3.48	0.232	5.98E-02	26.5	1.71E-06	1.28E-03
	std	0.42	0.285	5.77E+03	1.86E-18	0.517	0.111	1.35E-02	65.8	8.14E-07	7.99E-04
F7	avg	0	3.72E-03	6.96	1.71E-04	5.42E-03	0.183	0.121	6.28E-02	4.85E-04	5.99E-02
	std	0	4.51E-03	10.2	1.60E-04	3.10E-03	8.53E-02	3.71E-02	1.81E-02	8.45E-04	3.59E-02
Success		5/7	0/7	0/7	2/7	0/7	0/7	0/7	0/7	0/7	0/7

**Table 9:** Compared of mwoa and others for use on multimodal issues.

Function	Metric	MWOA-2	WOA	AC-WOA	SSA	GWO	WDO	PSO	DE	WOABAT	WOASAT
**F8**	**avg**	**− 1.26E+04**	**− 9.94E+03**	**− 8.57E+03**	**−3.18E+61**	**−40.1**	**−92.7**	**−1.53E+02**	**−7.10E+03**	**-1.22E+04**	**-9.97E+03**
	**std**	**2.89E+03**	**1.53E+03**	**2.15E+03**	**5.70E+61**	**6.71**	**1.10**	**37.0**	**1.13E+03**	**1.07E+03**	**1.65E+03**
**F9**	**avg**	**0**	**0**	**3.01E+02**	**3.43**	**27.9**	**60.7**	**19.0**	**1.65E+02**	**5.97**	**0**
	**std**	**0**	**0**	**42.7**	**9.29**	**22.4**	**32.5**	**4.89**	**25.4**	**11.9**	**0**
**F10**	**avg**	**0**	**3.82E-15**	**18.8**	**8.56E-15**	**6.67E-05**	**0.190**	**0.295**	**2.50**	**9.36E-04**	**8.88E−16**
	**std**	**0**	**2.38E-15**	**2.11**	**4.20E-14**	**6.94E-05**	**0.280**	**9.78E-02**	**3.36**	**2.11E-04**	**0**
**F11**	**avg**	**0**	**1.07E-02**	**75.2**	**0**	**1.29E-09**	**2.51E-03**	**3.17E-03**	**0.168**	**8.55E-08**	**0**
	**std**	**0**	**4.66E-02**	**61.3**	**0**	**2.56E-09**	**4.05E-03**	**1.02E-03**	**0.151**	**3.79E-08**	**0**
**F12**	**avg**	**8.00E-02**	**2.52E-02**	**3.51E+04**	**5.47E-32**	**0.263**	**3.58E-04**	**8.37E-04**	**1.18E+04**	**1.32E-08**	**1.81E-04**
	**std**	**4.67E-02**	**1.75E-02**	**1.03E+05**	**2.16E-31**	**6.22E-02**	**1.82E-04**	**1.40E-04**	**6.50E+04**	**5.60E-09**	**1.76E-04**
**F13**	**avg**	**1.08**	**0.428**	**4.74E+07**	**1.28E-17**	**2.15**	**8.30E-03**	**1.76E-02**	**4.50E+04**	**2.22E-07**	**1.35E-32**
	**std**	**0.276**	**0.263**	**1.42E+08**	**7.17E-17**	**0.256**	**1.40E-02**	**7.40E-03**	**1.80E+05**	**1.03E-07**	**5.47E-48**
**Success**		**4/6**	**1/6**	**0/6**	**2/6**	**0/6**	**0/6**	**0/6**	**0/6**	**0/6**	**3/6**

Significant values are in bold.

**Table 10:** Compared of mwoa and others for use on fixed- dimension multimodal issues.

Function	Metric	MWOA-2	WOA	AC-WOA	SSA	GWO	WDO	PSO	DE	WOABAT	WOASAT
F14	**avg**	**0.998**	**4.52**	**4.71**	**1.02**	**12.7**	**12.7**	**12.7**	**1.38**	**1.78**	**8.14**
	**std**	**3.67**	**4.18**	**4.69**	**6.62E-02**	**1.56E-10**	**1.40E-11**	**1.26E-12**	**1.77**	**2.50**	**5.09**
F15	**avg**	**0**	**6.47E-04**	**9.33E-02**	**1.66E-03**	**6.80E-04**	**4.47E-04**	**4.15E-04**	**1.36E-03**	**4.03E-04**	**5.51E-04**
	**std**	**0**	**2.36E-04**	**0.375**	**7.97E-19**	**4.48E-04**	**1.32E-04**	**1.42E-04**	**3.55E-03**	**3.56E-04**	**3.59E-04**
F16	**avg**	**-1.03**	**-1.03**	**-1.03**	**-1.01**	**-1.03**	**-1.03**	**-1.03**	**-1.03**	**-1.03**	**-1.03**
	**std**	**5.83E-10**	**7.81E-10**	**3.44E-04**	**2.03E-02**	**1.15E− 02**	**3.17E-04**	**2.51E-06**	**6.70E-16**	**5.53E-16**	**1.15E-10**
F17	**avg**	**0.397**	**0.397**	**1.15**	**0.418**	**0.759**	**0.397**	**0.548**	**0.397**	**0.3.97**	**0.397**
	**std**	**3.54E-04**	**7.15E-06**	**1.13**	**1.85E-02**	**1.19**	**2.32E-04**	**0.834**	**0**	**2.12E-15**	**8.79E-09**
F18	**avg**	**3.00**	**3.00**	**7.44**	**6.42**	**5.63**	**3.02**	**3.00**	**3.00**	**12.0**	**3.00**
	**std**	**9.65**	**1.61E-04**	**19.4**	**3.91**	**8.11**	**1.86E-02**	**5.67E− 05**	**2.33E-15**	**12.7**	**2.21E-08**
F19	**avg**	**-3.86**	**-3.86**	**-3.74**	**-3.86**	**-3.76**	**-3.86**	**-3.86**	**-3.86**	**-3.86**	**-3.86**
	**std**	**2.68E-02**	**6.56E-03**	**0.185**	**1.00E-02**	**0.535**	**6.28E-03**	**3.61E-03**	**2.70E-15**	**1.97E-03**	**0.193**
F20	**avg**	**-3.31**	**-3.18**	**-2.78**	**-2.83**	**-2.49**	**-3.09**	**-3.03**	**-3.25**	**-3.29**	**-3.26**
	**std**	**0.211**	**8.94E-02**	**0.406**	**0.112**	**0.918**	**0.203**	**0.363**	**5.77E-02**	**5.06E-02**	**5.93E-02**
F21	**avg**	**− 10.2**	**− 8.36**	**-3.71**	**− 9.01**	**− 4.78**	**− 5.03**	**-5.06**	**-9.99**	**-9.48**	**-5.40**
	**std**	**1.32**	**2.42**	**1.93**	**2.14**	**1.03**	**1.87E− 02**	**1.82E-04**	**0.906**	**1.7**	**1.27**
F22	**avg**	**− 10.4**	**− 7.77**	**-2.53**	**− 9.55**	**-4.68**	**-5.07**	**-5.09**	**-9.97**	**-9.17**	**− 5.09**
	**std**	**2.69**	**2.97**	**1.56**	**1.96**	**1.24**	**1.65E-02**	**3.24E-04**	**1.66**	**2.24**	**2.26E-07**
F23	**avg**	**− 10.5**	**− 7.21**	**− 2.20**	**− 8.98**	**− 4.75**	**− 5.10**	**− 5.13**	**− 9.88**	**− 1.00**	**− 5.31**
	**std**	**3.14**	**3.39**	**1.01**	**2.46**	**1.16**	**1.96E-02**	**2.28E− 04**	**2.05**	**1.61**	**0.971**
Success		**10/10**	**4/10**	**1/10**	**1/10**	**1/10**	**3/10**	**3/10**	**4/10**	**3/10**	**4/10**

Significant values are in bold.

#### Analysis of exploitation

The unimodal functions (F1–F7) get measured by employing an optimizer’s exploitation ability. Table 8 displays the findings from the optimizer evaluations. Table 6's highlights reflect the optimal outcomes. Based on the findings in Table 5, among essential optimizers (WOA, SSA, GWO, WDO, PSO, and DE), the performance of SSA is better than others only in (F5, F6). When compared to MWOA, it offers the greatest optimization performance for F1-F4 and F7, demonstrating its superior local exploitation stability and power.

To be more explicit, the random evolutionary approach allows the algorithm to swiftly explore a search region, and the specific reinforcement of a design technique can aid in obtaining the best solution through additional exploitation of a discovered region.

#### Analysis of exploration

When contrasted with unimodal functions, multimodal functions comprise a high number of local optima, no. of which grows larger with the complexity of the problem. As a result, these types of issues are often used to test an algorithm’s exploration capacity. Table 9 demonstrates that, out of the 6 functions tested, MWOA-2 has significantly higher competitiveness than others in 4 of them. The best mean value and STD determined by MWOA-2, WOA, SSA, and WOASAT are in (F9, F10, F11), (F9), (F11), and (F9, F11) respectively. However, WOASAT outperforms SSA only for F13, SSA outperforms MWOA-2 only for F12, and MWOA-2 outperforms them all for F8 functions. As a result, it is determined that MWOA-2 outperforms them for multimodal functions. It demonstrates how MWOA-2 may investigate the region with greater properly and reliably, as well as avoid local optima.

This pertains to adaptive crossover, and an enhanced step-size factor increases the population’s variety and randomness. Furthermore, as shown in Table 10, optimizing the F14–F23 is relatively straightforward due to its low dimensionality, resulting in only minor differences in optimum outcomes for certain functions. In reality, MWOA-2 proves to be a top or second-best optimizer in most benchmark functions, as indicated by the statistical results. These findings demonstrate that MWOA-2 can consistently explore the unidentified solution region, thereby increasing the likelihood of escaping local optima.

#### Analysis of convergence

Figure 7 depicts the converging curves for 23 typical benchmark issues used to assess the convergence of MWOA-2. X_axis inside the graphs = the no. of iterations. Y_axis = best score obtained so far. When it comes to unimodal functions, MWOA-2 outperforms the others.

MWOA-2 may rapidly converge to an optimum solution for F1, F2, F5, F6, and F7 in the early stages, while other methods converge more slowly. Functions F3 and F4 reveal that, like WOA, AC-WOA, DE, and PSO, MWOA-2 initially descends towards local solutions during the convergence process, but uniquely, MWOA-2 resists getting trapped in local minima and reaches the global minimum. It demonstrates how MWOA-2 generates higher-quality solutions. The capacity to prevent local minima is attributed to the random evolutionary technique, enabling the algorithm to accumulate knowledge about superior solutions, akin to top whales, thereby narrowing the population’s search range in the early stages. The convergence results of these unimodal functions demonstrate how the suggested technique significantly enhances both convergence speed and accuracy. MWOA-2 exhibits the ability to maintain stable convergence speed and accuracy.

With F9-F10, MWOA-2 can rapidly achieve the global optimal solution, while AC-WOA, DE, WDO, and PSO converge slowly, resulting in low-precision results. MWOA-2 divides whale hunting techniques for early and late phases using a weighting factor. Despite MWOA-2's slower convergence speed in functions F11 and F13 compared to AC-WOA, it can provide superior quality solutions. This demonstrates that the optimizer possesses strong continuous development abilities and is capable of discovering and exploiting the best value. Most fixed-dimension multimodal convergence curves are essentially comparable.

When compared to WOA, GWO, WOA-BAT, WOA-SAT, SSA, and DE in the fixed-dimension multimodal scenario, MWOA-2's convergence speed and accuracy are comparable. In summary, MWOA-2's convergence performance indicates that randomized evolutionary and specific reinforcing mechanisms can assist the optimizer in discovering the optimum solution in the solution area more efficiently.

As a result, findings of this part show that the efficiency of the suggested algorithm has increased. Enhanced exploration capabilities of MWOA-2 are attributed to the improved whale location update technique that utilizes Eq. (5). This equation needs whales to maneuver increasingly irregularly into one another and rapidly increase their gap. Furthermore, increased exploitation and convergence are highlighted as a result of Eq. (10). This mathematics enables the whales to quickly adjust themselves in accordance with the ideal solution. Simultaneously, whales use Eq. (12) to ensure population variety and prevent local optima.

Based on the results of the preceding investigation, we describe the limitations and highlights of the suggested algorithm. The features of MWOA-2 include double the chassis repairs and an update method for the placement of the underlying algorithm. The growth in the algorithm’s structure goes beyond a basic replacement; it involves a rethinking of operational guidelines and regulations. In certain fixed-dimension multimodal situations, MWOA-2 does have a few limitations, such as low accuracy and speed.

### Experiment number 5

**Fig. 8:**
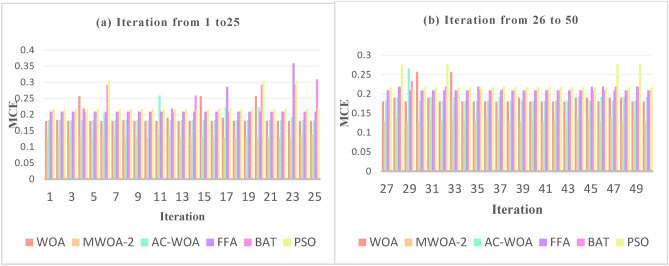
Numerous rounds to a train utilizing a distinct set for selected features “hypertensive patient”.

**Fig. 9:**
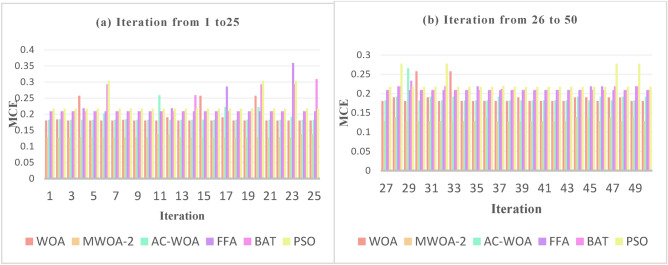
Numerous rounds to a train utilizing a distinct set for selected features " hypotensive patient".

**Fig. 10:**
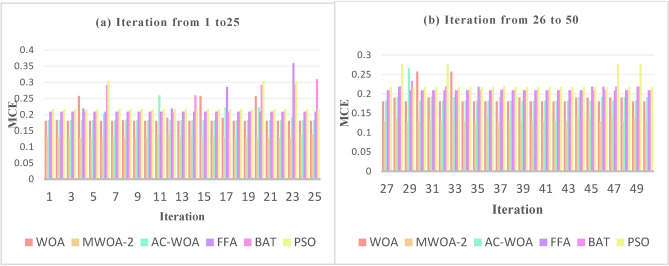
Numerous rounds to a train utilizing a distinct set for selected features " normotensive patient".

The MCE for WOA, AC-WOA, FFA, BAT, and PSO optimizers is used to compare the performance of MWOA-2. The experiment is designed to demonstrate that the recommended strategy enhances performance. Over 50 iterations, the findings are displayed in the figures 8, 9, and 10 below. These numbers illustrate the supremacy of MWOA-2 over others.

### Experiment number 6

**Fig. 11:**
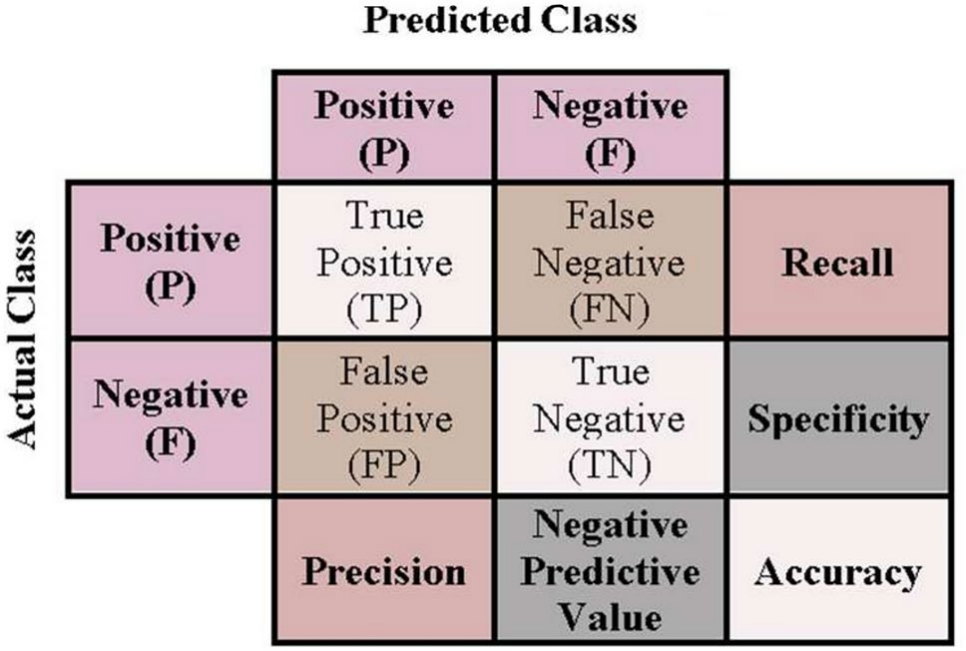
The confusion matrix.

In this section, the MWOA-2 is tested against WOA, AC-WOA, FFA, BAT, and PSO algorithms to verify its performance. The confusion matrix (Figure 11) is utilized here. One essential tool for evaluating the effectiveness of deep learning frameworks. Additionally, several important metrics, as defined by EQs. (21–23), such as recall, accuracy, and precision, are calculated. The efficacy of the suggested framework can be more precisely and subtly evaluated thanks to these measures.

True Positive (TP): is the no. correct positive predictions. False Negative (FN): is the no. incorrect negative predictions. False Positive (FP): is the no. incorrect positive predictions. True Negative (TN): is the no. correct negative predictions.

21 $$Accuracy= \frac{TP+TN}{(TP+FN+FP+TN)}$$

22 $$Precision= \frac{TP}{(TP+FP)}$$

23 $$Recall= \frac{TP}{(TP+FN)}$$

The findings are displayed in Table 11 below. These numbers illustrate the supremacy of MWOA-2 over others.

**Table 11:** Compared mwoa-2 and others in terms of accuracy, precision, and recall.

	Dataset	MWOA-2	AC-WOA	WOA	FFA	BAT	PSO
Accuracy	P1	**98%**	93.6%	92.5%	63.7%	78.8%	87.85%
	P2	**94.4%**	92.46%	93%	75.07%	79.1%	45.6%
	P3	**95.38%**	91.8%	94.7%	81.61%	74.3%	67.7%
Precision	P1	**87.5%**	70.5%	71.5%	63.7%	78.8%	39.38%
	P2	**94.9%**	84.48%	70.2%	68.77%	84.7%	66.5%
	P3	**95.32%**	84.4%	80.9%	81.9%	83.4%	68.6%
Recall	P1	**88.4%**	72%	71.5%	63.7%	78.8%	50.1%
	P2	**94.9%**	70.66%	68.4%	68.77%	80.4%	69.1%
	P3	**97.49%**	73.9%	72.1%	81.89%	95.3%	74.95%

Significant values are in bold.

## Conclusion and future directions

Healthcare systems handle vast amounts of data, yet there is a shortage of effective analytic techniques to unveil the underlying linkages in this data. The proposed R-DLH2O framework is utilized for predicting emergency cardiovascular situations among the elderly. It comprises 5 phases: a robust pre-processing phase, FS phase, optimized FFNN phase, prediction phase, and performance evaluation phase. The Modified WOA (MWOA) is a novel version of WOA tailored for use with R-DLH2O to enhance its performance.

Three public datasets were employed in 6 experiments to validate the R-DLH2O Framework. MWOA, WOA, PSO, FFA, AC-WOA, and BAT were all compared. MWOA achieved an average mean per-class error of 0.150125 with an average processing time of 436 seconds, an accuracy 95.93%, a precision 92.57%, and a recall 93.6% across all datasets. The strengths and limitations of the new technique were evident, as it surpassed the original whale optimizer in terms of scalability and speed and demonstrated greater accuracy compared to the majority of the examined algorithms.

Future comparisons will involve new meta-algorithms against our suggested WOA with the implementation of this proposed model with different sampling techniques^56,57^ to improve the performance. Additionally, ongoing research is investigating the impact of increasing the complexity of data used in DL.

## Data Availability

The datasets analyzed during the current study are available in the [PhysioNet MIMIC-II] repository, https://physionet.org/content/mimic-ii/2.6.0/

## References

[1] Ding, X. R. et al. Continuous cuffless blood pressure estimation using pulse transit time and photoplethysmogram intensity ratio. *IEEE Trans. Biomed. Eng. ***63**(5), 964–972 (2015). 26415147 10.1109/TBME.2015.2480679

[2] Höcht, C. “Blood pressure variability: prognostic value and therapeutic implications”. *ISRN hypertension*, pp. 1–16(2013).

[3] El-Hajj, C and P a Kyriacou. A review of machine learning techniques in photoplethysmography for the non-invasive cuff-less measurement of blood pressure”. *Biomedical Signal Processing and Control* 58(2020).

[4] El-Hajj, C and P a Kyriacou. “Deep learning models for cuffless blood pressure monitoring from PPG signals using attention mechanism, *Biomed. Sig. Process. Control* 65(2021).

[5] Higgins, J. R. & Swiet, M. Blood-pressure measurement and classification in pregnancy. *Lancet ***357**(9250), 3552–3554 (2001). 10.1016/S0140-6736(00)03552-2 11197413

[6] Parati, G. et al. Blood pressure variability: clinical relevance and application. *J. Clin. Hypertens. ***20**(7), 1133–1137 (2018). 10.1111/jch.13304 PMC8030809 30003704

[7] O’brien, E, et al. European Society of Hypertension recommendations for conventional, ambulatory and home blood pressure measurement. *J. Hypertens. ***21**(5), 821–848 (2003). 12714851 10.1097/00004872-200305000-00001

[8] Ogunpola, A. 2024 Machine Learning-Based Predictive Models for Detection of Cardiovascular Diseases. *Diagnostics ***14**, 144–144 (2024). 38248021 10.3390/diagnostics14020144 PMC10813849

[9] Degroat, W., Abdelhalim, H. & Patel, K. Discovering biomarkers associated and predicting cardiovascular disease with high accuracy using a novel nexus of machine learning techniques for precision medicine. *Sci. Rep. ***14**, 1–1 (2024). 38167627 10.1038/s41598-023-50600-8 PMC10762256

[10] documentation, H2O.ai H2O, ed., pp. 13–13. URL: https://docs.h2o.ai/,2024. (2024)

[11] Bengio, Y. Learning deep architectures for AI. *Foundations and trends® in Machine Learning*, 2(1), 1–127(2009).

[12] El-Ghamrawy, S M, A I El-Desouky, and M Sherief. Dynamic ontology mapping for communication in distributed multi-agent intelligent system. *2009 International Conference on Netw. Media Converg. ***1**, 103–108 (2009).

[13] El-Ghamrawy, S. M. & Eldesouky, A. I. An agent decision support module based on granular rough model. *Int. J. Inf. Technol. Decis. Making*10.1142/S0219622012500216 (2012).

[14] El-Ghamrawy, S. M. & El-Desouky, A. I. Distributed multi-agent communication system based on dynamic ontology mapping. *Int .J. Commun. Netw. Distr. Syst. ***10**(1), 1–24 (2013).

[15] Tan, K. C. et al. Balancing exploration and exploitation with adaptive variation for evolutionary multi-objective optimization. *Eur. J. Oper. Res.*10.1016/j.ejor.2008.07.025 (2009).

[16] Mafarja, M. M. & Mirjalili, S. Hybrid whale optimization algorithm with simulated annealing for feature selection. *Neurocomputing*10.1016/j.neucom.2017.04.053 (2017).

[17] Ding, Y., Zhou, K. & Bi, W. Feature selection based on hybridization of genetic algorithm and competitive swarm optimizer. *Soft Comput. ***24**(15), 11663–11672 (2020).

[18] Kahya, M. A., Altamir, S. A. & Algamal, Z. Y. Improving whale optimization algorithm for feature selection with a time-varying transfer function numerical algebra. *Control Optim.*10.3934/naco.2020017 (2021).

[19] Hsu, H. H., Hsieh, C. W. & Lu, M. D. Hybrid feature selection by combining filters and wrappers. *Expert Syst. Appl. ***38**(7), 8144–8150 (2011).

[20] Butler-Yeoman, T, B Xue, and M Zhang .“Particle swarm optimisation for feature selection: A hybrid filter-wrapper approach”. *2015 IEEE congress on evolutionary computation (CEC) ***1**, 2428–2435 (2015).

[21] Han, H., Wang, W.-Y. & Mao, B.-H. Borderline-smote: a new oversampling method in imbalanced data sets learning. *Int. Conf. Intell. Comput.*10.1007/11538059_91 (2005).

[22] He, H et al. “Adasyn: Adaptive synthetic sampling approach for imbalanced learning”. *IEEE international joint conference on neural networks ***1**, 1322–1328 (2008).

[23] Elseddeq, N. G. et al. A selected deep learning cancer prediction framework”. *IEEE Access*10.1109/ACCESS.2021.3124889 (2021).

[24] Khan, W. A., Chung, S. H., Awan, M. U. & Wen, X. Machine learning facilitated business intelligence (Part II) Neural networks optimization techniques and applications. *Indus. Manag. Data Syst. ***120**(1), 128–163 (2020).

[25] Mahmud, S et al. “A shallow U-Net architecture for reliably predicting blood pressure (BP) from photoplethysmogram (PPG) and electrocardiogram (ECG) signals”. *Sensors ***22**(3), 919 (2022). 10.3390/s22030919 PMC8840244 35161664

[26] Alam, S., Gupta, R. & Sharma, K. D. On-board signal quality assessment guided compression of photoplethysmogram for personal health monitoring. *IEEE Trans. Instrum. Measur.*10.1109/TIM.2021.3067238 (2021).

[27] Paviglianiti, A. et al. A comparison of deep learning techniques for arterial blood pressure prediction. *Cogn. Comput.*10.1007/s12559-021-09910-0 (2022). 10.1007/s12559-021-09910-0 PMC8391010 34466163

[28] Prasun, P., Mukhopadhyay, S. & Gupta, R. Real-time multi-class signal quality assessment of photoplethysmography using machine learning technique. *Measur. Sci. Technol. ***33**(1), 15701–15701 (2021).

[29] Yin, Y. & Chou, C. A. A novel switching state-space model for post-icu mortality prediction and survival analysis”. *IEEE J. Biomed. Health Inform. ***25**(9), 3587–3595 (2021). 33755571 10.1109/JBHI.2021.3068357

[30] Neha et al. “Arrhythmia detection and classification using ECG and PPG techniques: A review”. *Physical and Engineering Sciences in Medicine*, 1–22(2021). 10.1007/s13246-021-01072-5 34727361

[31] Reddy, G N K, M S Manikandan, and N N Murty. “Lightweight compressed sensing (CS) and partial DCT based compression schemes for energy-efficient wearable PPG monitoring devices”. *2021 IEEE International Conference on Health, Instrumentation & Measurement, and Natural Sciences (InHeNce)*, pp. 1–6(2021).

[32] Nakamura, R Y et al. “BBA: a binary bat algorithm for feature selection”. *2012 25th SIBGRAPI conference on graphics, patterns and images*, 291–297(2012).

[33] Sharawi, M, H M Zawbaa, and E Emary. “Feature selection approach based on whale optimization algorithm”. *2017 Ninth international conference on advanced computational intelligence (ICACI)*, 163–168(2017).

[34] Hassan, M. K. et al. A Hybrid Real-time remote monitoring framework with NB-WOA algorithm for patients with chronic diseases. *Fut. Gener. Comput. Syst. ***93**, p77-95 (2019).

[35] Mirjalili, S. & Lewis, A. The whale optimization algorithm. *Adv. Eng. Softw. ***95**, 51–67 (2016).

[36] Mejía-Mejía, E et al. “Differential effects of the blood pressure state on pulse rate variability and heart rate variability in critically ill patients”. *NPJ digital medicine ***4**(1), 82 (2021). 10.1038/s41746-021-00447-y PMC8121822 33990692

[37] Harfiya, L. N., Chang, C. C. & Li, Y. H. Continuous blood pressure estimation using exclusively photopletysmography by LSTM-based signal-to-signal translation”. *Sensors ***21**(9), 2952–2952 (2021). 33922447 10.3390/s21092952 PMC8122812

[38] Li, P. & Laleg-Kirati, T. M. Central blood pressure estimation from distal PPG measurement using semiclassical signal analysis features. *IEEE Access ***9**, 44963 (2021).

[39] Elghamrawy, S. M. & Hassanien, A. E. A hybrid Genetic-Grey Wolf Optimization algorithm for optimizing Takagi-Sugeno-Kang fuzzy systems. *Neural Comput. Appl. ***34**(19), 17051–17069 (2022).

[40] Elghamrawy, S M, A E Hassanien, and A V Vasilakos. Genetic-based adaptive momentum estimation for predicting mortality risk factors for COVID-19 patients using deep learning. *Int. J. Imaging Syst. Technol. ***32**(2), 614-628 (2022). 10.1002/ima.22644 PMC8426801 34518740

[41] Khan, W. A., Chung, S. H., Awan, M. U. & Wen, X. Machine learning facilitated business intelligence (Part I) Neural networks learning algorithms and applications. *Indus. Manag. Data Syst. ***120**(1), 164–195 (2020).

[42] Elsayad, A. S., El Desouky, A. I., Salem, M. M. & Badawy, M. A deep learning H 2 O framework for emergency prediction in biomedical big data. *IEEE Access. ***8**, 97231–97242 (2020).

[43] Bhanja, S., & Das, A. Impact of data normalization on deep neural network for time series forecasting. 10.48550/arXiv.1812.05519 (2018).

[44] Hulse, J Van, T M Khoshgoftaar, and A Napolitano. “Experimental perspectives on learning from imbalanced data”. In: *Proc. 24th international conference on machine learning, ICML ’07*, association for computing machinery, pp. 935–942(2007).

[45] Krawczyk, B. Learning from imbalanced data: open challenges and future directions. *Prog. Artif. Intell. ***5**(4), 221–253 (2016).

[46] Pal, S. K. & Anand, S. Cryptography based on RGB color channels using ANNs”. *Int. J. Comput. Netw. Inform. Sec.*10.5815/ijcnis.2018.05.07 (2018).

[47] Sree Kala, T. & Christy, A. HFFPNN classifier: a hybrid approach for intrusion detection based OPSO and hybridization of feed forward neural network (FFNN) and probabilistic neural network (PNN). *Multimed. Tools Appl. ***80**(4), 6457–6478 (2021).

[48] El-Kenawy, E. S. M. et al. MbGWO-SFS: Modified binary grey wolf optimizer based on stochastic fractal search for feature selection. *IEEE Access*10.1109/ACCESS.2020.3001151 (2020).34976558

[49] Moody, G. B., Mark, R. G. & Goldberger, A. L. PhysioNet: a web-based resource for the study of physiologic signals. *IEEE Eng. Med. Biol. Magazine ***20**(3), 70–75 (2001). 10.1109/51.932728 11446213

[50] Saeed, M. et al. Multiparameter Intelligent Monitoring in Intensive Care II (MIMIC-II): a public-access intensive care unit database”. *Crit. Med.*10.1097/CCM.0b013e31820a92c6 (2011). 10.1097/CCM.0b013e31820a92c6 PMC3124312 21283005

[51] Jia, L., Gong, W. & Wu, H. An improved self-adaptive control parameter of differential evolution for global optimization. In *Computational Intelligence and Intelligent Systems: 4th International Symposium* (eds Cai, Z. et al.) (Springer, 2009).

[52] Jain, M., Singh, V. & Rani, A. A novel nature-inspired algorithm for optimization: Squirrel search algorithm. *Swarm Evol. Comput. ***44**, 148–175 (2019).

[53] Mirjalili, S., Mirjalili, S. M. & Lewis, A. Grey wolf optimizer. *Adv. Eng. Softw. ***69**, 46–61 (2014).

[54] Bayraktar, Z, M Komurcu, and D H Werner. “Wind Driven Optimization (WDO): A novel nature-inspired optimization algorithm and its application to electromagnetics”. *2010 IEEE antennas and propagation society international symposium*, pp. 1–4. 10.1109/APS.2010.5562213 (2010).

[55] Das, S. & Suganthan, P. N. Differential evolution: A survey of the state-of-the-art”. *IEEE Trans. Evol. Computat. ***15**, 4 (2010).

[56] Khan, W. A. Balanced weighted extreme learning machine for imbalance learning of credit default risk and manufacturing productivity. *Ann. Oper. Res.*10.1007/s10479-023-05194-9 (2023).

[57] Khan, W. A., Chung, S. H., Eltoukhy, A. E. & Khurshid, F. A novel parallel series data-driven model for IATA-coded flight delays prediction and features analysis. *J. Air Trans. Manag. ***114**, 102488 (2024).

